# Supplementary material for: Sensitive extraction-free SARS-CoV-2 RNA virus detection using a novel RNA preparation method
Source: medRxiv. 2021 Feb 1:2021.01.29.21250790. Preprint. [Version 1] doi: 10.1101/2021.01.29.21250790 (PMC7852279; doi:10.1101/2021.01.29.21250790)

medRxiv preprint doi: https://doi.org/10.1101/2021.01.29.21250790; this version posted February 1, 2021. The copyright holder for this preprint (which was not certified by peer review) is the author/funder, who has granted medRxiv a license to display the preprint in perpetuity.
This article is a US Government work. It is not subject to copyright under 17 USC 105 and is also made available for use under a CC0 license.

Figure S1. DMSO decreases negative droplet intensity in RT-ddPCR assays. The RT-ddPCR reactions containing 0%, 2.5%, or 5% DMSO were performed for a Chelex-lowTE sample prepared with the ATCC SARS-CoV-2 virions and 293FT cells using N1 and cRPP30 (left panel) or N2 and RPP30 (right panel). The grey clusters represent negative droplets; blue and green clusters represent Fam and Hex positive droplets, respectively; and orange clusters represent double positive droplets.

Figure S2. SARS-CoV-2 prepared in different buffers used for RT-qPCR without RNA-extraction. **(A)** Samples were diluted in H_2O as indicated at the bottom. Expected_Ct refers to Ct calculated based on Ct from extracted RNA normalized with added virion numbers after dilution using the ΔCt method. **(B)** Buffer compatibility in RT-qPCR. Sample RNA, not heated and 5 µl of which contained materials extracted from 6,250 virions. Other samples were heated in the presence of Chelex, of which undiluted samples also contained 6,250 virions per 5 µl. Samples were diluted in H_2O. Samples with undetermined Ct values were plotted as Ct 40. The NEB Luna RT-qPCR kit and NEB-Luna-Program I was used. NTC, no-template control.

Figure S3. Tris EDTA and DMSO containing buffers. **(A)** RT-qPCR of samples with heat-inactivated ATCC SARS-CoV-2 virions. 5 µl of samples were used for one reaction in RT-qPCR except that samples in MEM α were diluted 1:1 with H_2O. Samples with undetermined Ct values were plotted as Ct 40. The NEB Luna RT-qPCR kit and NEB-Luna-Program I was used. **(B)** RT-ddPCR of saliva samples with heat-inactivated ATCC SARS-CoV-2 virions. The Chelex was prepared in H_2O, lowTE or TED99

medRxiv preprint doi: https://doi.org/10.1101/2021.01.29.21250790; this version posted February 1, 2021. The copyright holder for this preprint (which was not certified by peer review) is the author/funder, who has granted medRxiv a license to display the preprint in perpetuity. This article is a US Government work. It is not subject to copyright under 17 USC 105 and is also made available for use under a CC0 license.

(lowTE with 99% DMSO). RNA-kit refers to RNA extracted with the RNeasy Protect Saliva Mini Kit. NTC, no-template control.

Figure S4. Optimization of the NEB Luna RT-qPCR assay. Extracted RNA samples were serial diluted and assayed either using 2.5 µl sample in a 10 reaction volume or 5 µl in a 20 µl reaction, and using a longer PCR protocol (I: 10 seconds of denature and 40 seconds of annealing/extension) or a shorter PCR protocol (II: 5 seconds of denature and 20 seconds of annealing/extension). NTC, no-template control.

medRxiv preprint doi: https://doi.org/10.1101/2021.01.29.21250790; this version posted February 1, 2021. The copyright holder for this preprint (which was not certified by peer review) is the author/funder, who has granted medRxiv a license to display the preprint in perpetuity.
This article is a US Government work. It is not subject to copyright under 17 USC 105 and is also made available for use under a CC0 license.

Figure S1

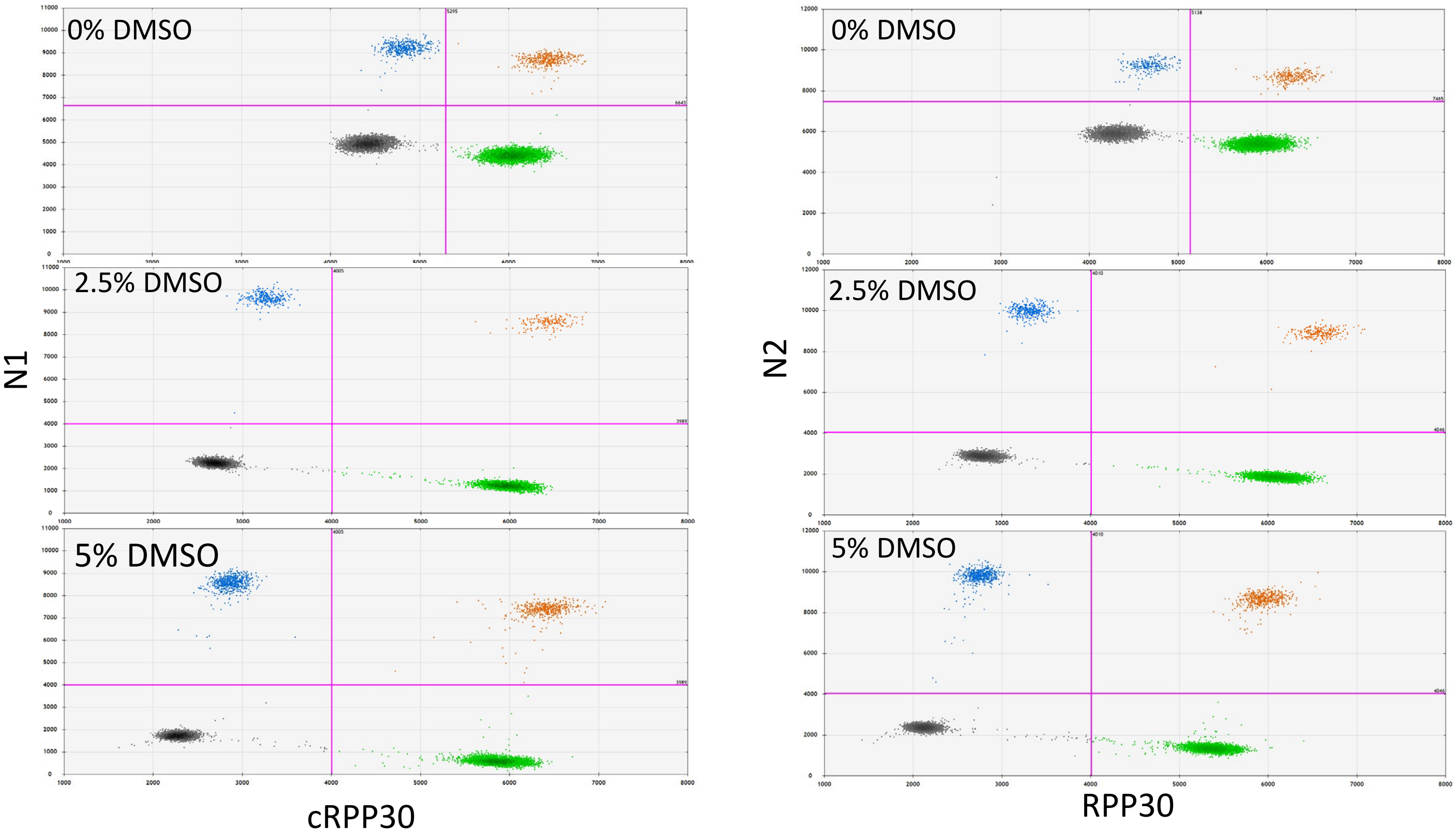

medRxiv preprint doi: https://doi.org/10.1101/2021.01.29.21250790; this version posted February 1, 2021. The copyright holder for this preprint (which was not certified by peer review) is the author/funder, who has granted medRxiv a license to display the preprint in perpetuity.
This article is a US Government work. It is not subject to copyright under 17 USC 105 and is also made available for use under a CC0 license.

Figure S2

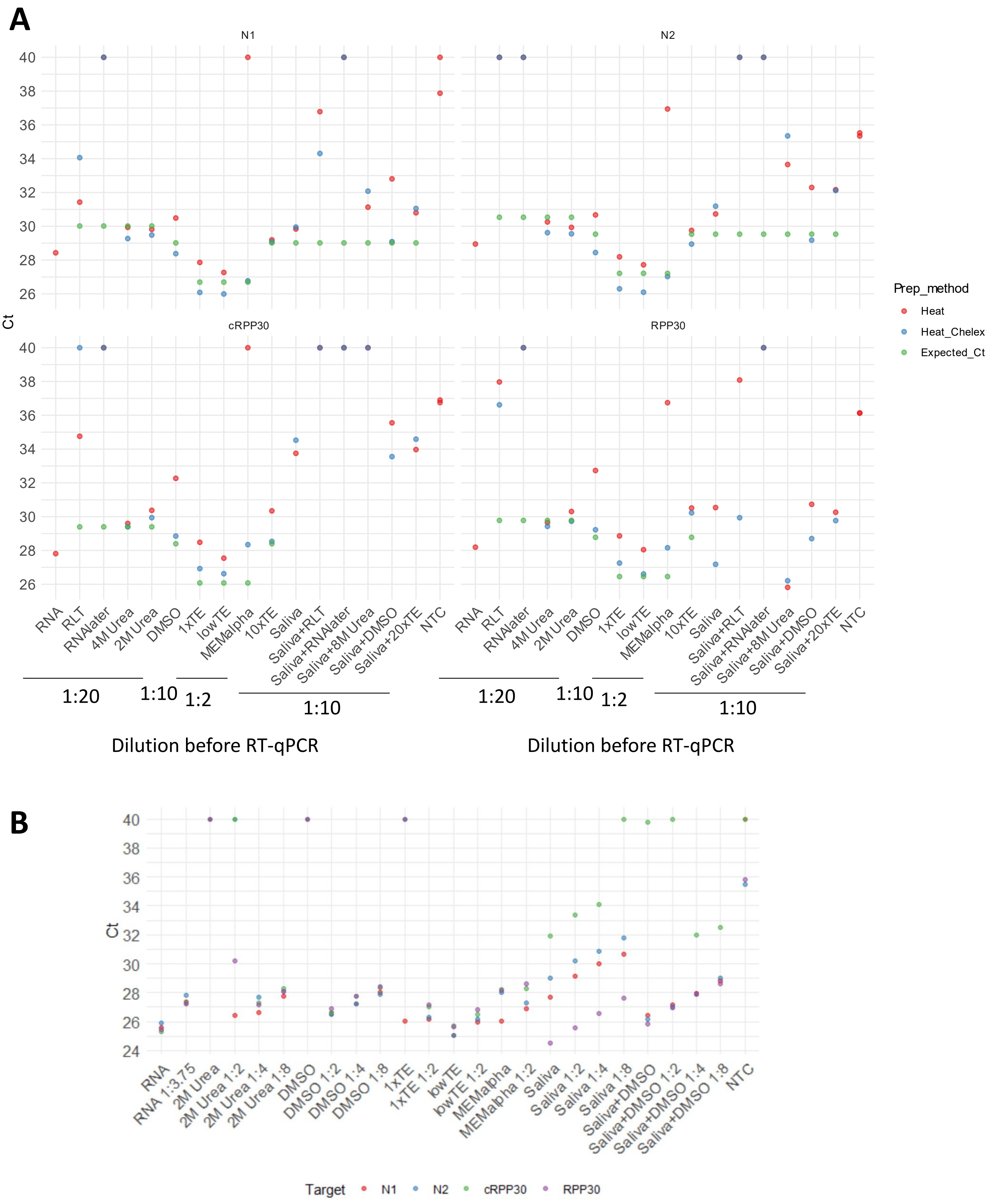

medRxiv preprint doi: https://doi.org/10.1101/2021.01.29.21250790; this version posted February 1, 2021. The copyright holder for this preprint
(which was not certified by peer review) is the author/funder, who has granted medRxiv a license to display the preprint in perpetuity.
This article is a US Government work. It is not subject to copyright under 17 USC 105 and is also made available
for use under a CC0 license.

Figure S3

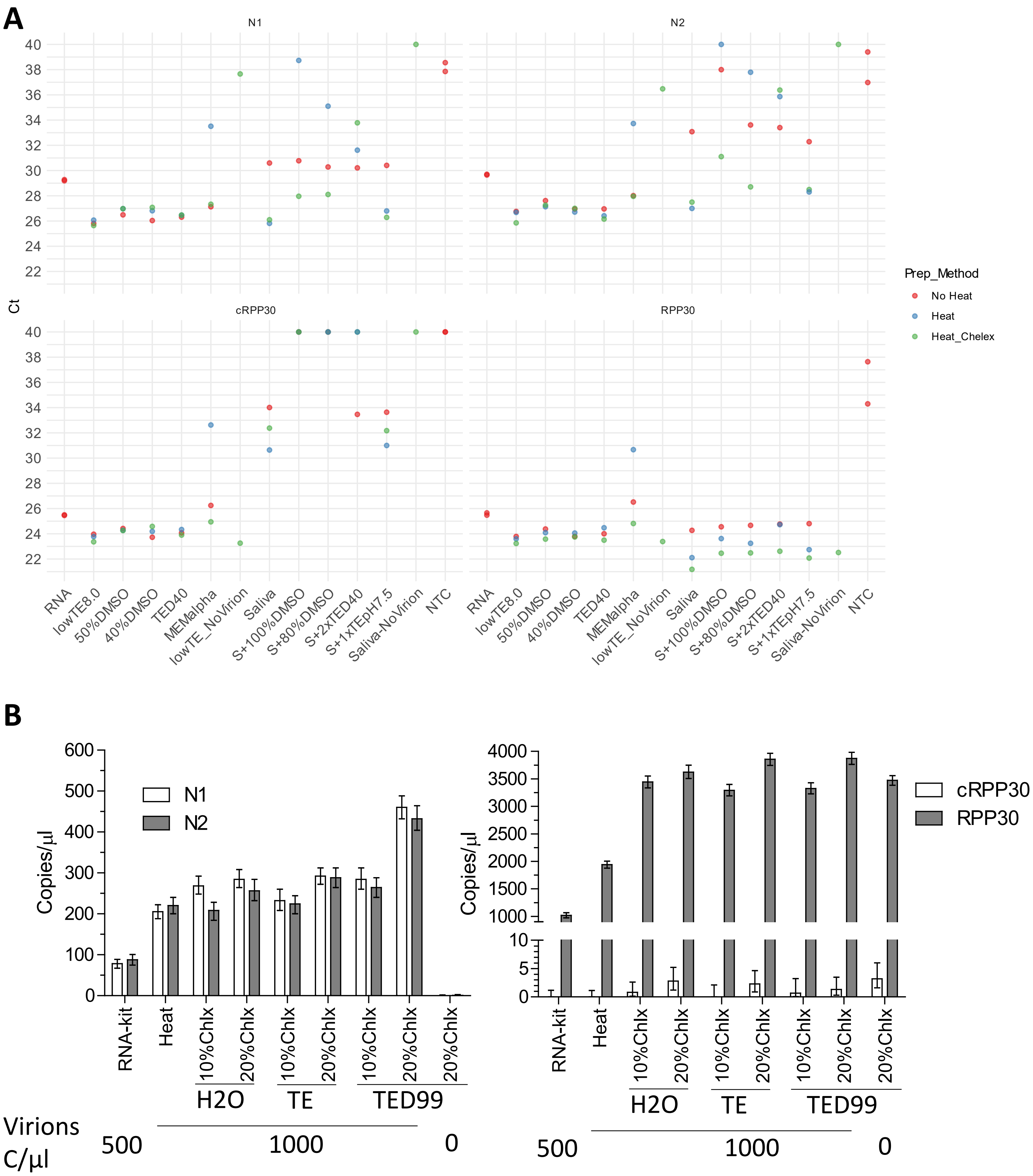

medRxiv preprint doi: https://doi.org/10.1101/2021.01.29.21250790; this version posted February 1, 2021. The copyright holder for this preprint
(which was not certified by peer review) is the author/funder, who has granted medRxiv a license to display the preprint in perpetuity.
This article is a US Government work. It is not subject to copyright under 17 USC 105 and is also made available
for use under a CC0 license.

Figure S4

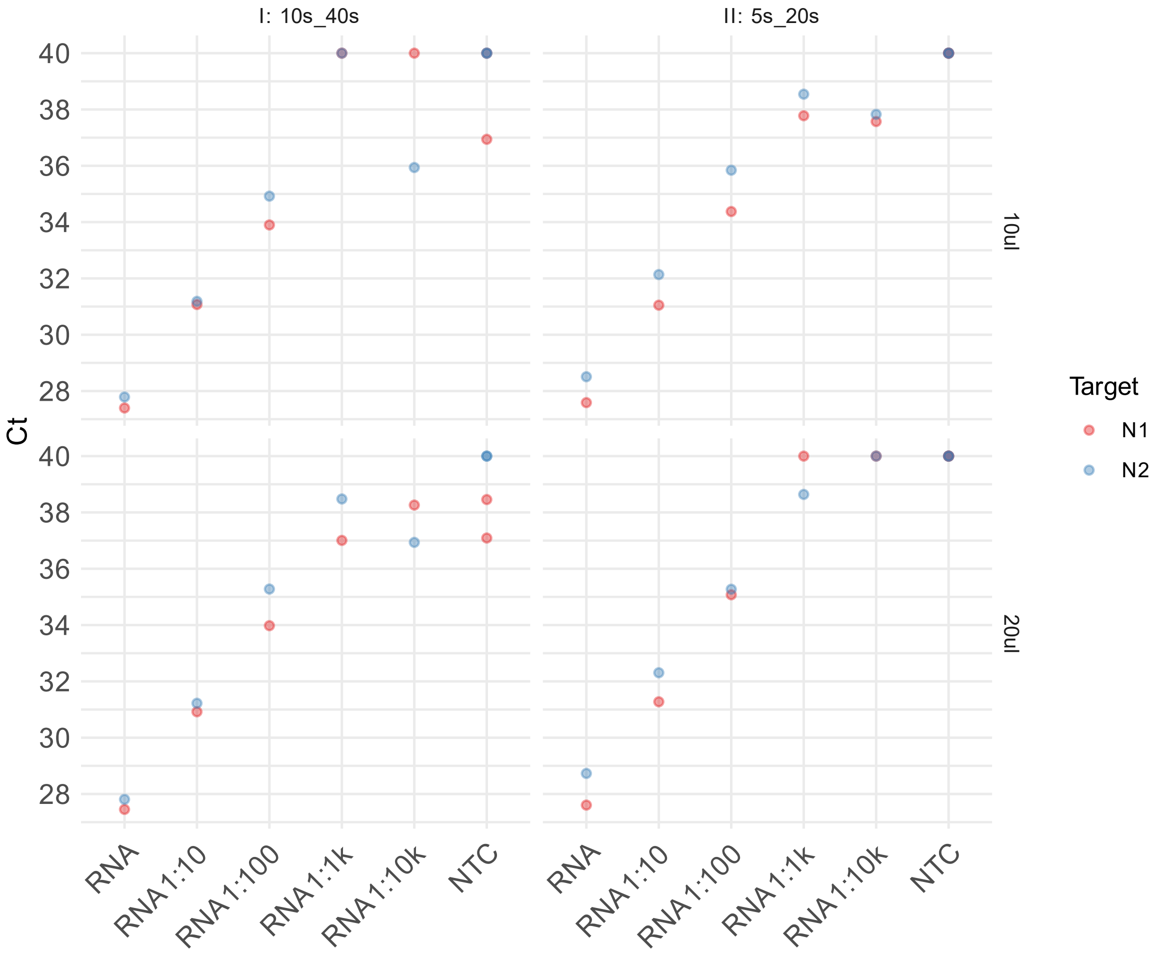

Supplement: 1 [file NIHPP2021.01.29.21250790-supplement-1.pdf]
